# 2889. One vs Three Weekly Doses of Benzathine Penicillin G for Treatment of Early Syphilis in Persons with and without HIV: A Multicenter Randomized Controlled Trial (RCT)

**DOI:** 10.1093/ofid/ofad500.2472

**Published:** 2023-11-27

**Authors:** Edward W Hook, Kimberly Workowski, Jodie A Dionne, Candice J McNeil, Stephanie N Taylor, Batteiger Teresa, Julia C Dombrowski, Kenneth H Mayer, Arlene C Seña, Harold C Wiesenfeld, Charlotte Perlowski, Lori Newman, Chunming Zhu, Jorge E Mejia-Galvis

**Affiliations:** University of Alabama at Birmingham, Birmingham, AL; Emory University, Atlanta, Georgia; University of Alabama at Birmingham, Birmingham, AL; Wake Forest School of Medicine, Winston Salem, NC; Louisiana State University Health Sciences Center, New Orleans, Louisiana; Indiana University School of Medicine, Indianapolis, Indiana; University of Washington, Seattle, Washington; Harvard Medical School/Fenway Research Institute, Boston, MA; Division of Infectious Diseases, Department of Medicine, University of North Carolina at Chapel Hill School of Medicine, Chapel Hill, NC; University of Pittsburgh School of Medicine, Pittsburgh, Pennsylvania; FHI 360, Durham, North Carolina; NIH/NIAID, Rockville, Maryland; The Emmes Company, LLC, Rockville, Maryland; NIAID/NIH, Rockville, Maryland

## Abstract

**Background:**

Syphilis rates in the United Stated (U.S.) have increased steadily for over a decade. Despite over 75 years as the drug of choice for syphilis treatment, controversy persists on optimal duration of therapy with benzathine penicillin G (BPG) for persons with early(primary, secondary, and early latent) syphilis, particularly for persons co-infected with HIV. Given the ongoing national shortages of BPG, optimizing duration of therapy is an urgent concern.

**Methods:**

We conducted a multicenter RCT comparing a single intramuscular (IM) injection of BPG, 2.4 million units to BPG administered for three successive weeks for treatment of early syphilis in persons with and without HIV. The primary outcome of the study was a > 4-fold decline in RPR titer measured at 6 months. Intention to treat (ITT) and per protocol analyses were performed and were similar. ITT analyses are presented here.

**Results:**

A total of 249 persons with early syphilis were enrolled at 10 participating sites. Most (97%) participants were were categorized as male sex, black race (62%), and 153 (64%) were living with HIV. The syphilis stage distribution was 19% primary, 47% secondary, and 33% early latent and did not differ significantly by HIV status. Serologic response (> 4-fold decline in RPR titer) at 6 months was 76% (95% Confidence Interval (CI) 0.68-0.82) in the single dose group and did not differ significantly from the three-dose group (70%, 95% CI 0.61-0.77). There were no treatment failures (> 4-fold increase in RPR titer). Among persons with and without HIV there was no significant difference in RPR response at 6 months: 76% in the single-dose group vs. 71% in the 3-dose group (95% CI 0.05-0.17). Most participants experienced mild to moderate local injection site pain and tenderness.

**Conclusion:**

Treatment of persons with early syphilis with more than a single dose of 2.4 million units of BPG offers no therapeutic benefit irrespective of HIV infection status and was associated with increased rates of injection site discomfort.

**Disclosures:**

**Edward W. Hook, III, FIDSA**, GARPD (Global Antibiotic Resistance Program: Advisor/Consultant|Talis Biomedical: Advisor/Consultant|VISBY Diagnostics: Advisor/Consultant **Candice J. McNeil, MD, MPH**, BARDA/GSK: Grant/Research Support|Becton Dickinson: Grant/Research Support|Cepheid: Grant/Research Support|Gilead: Grant/Research Support|Hologic: Grant/Research Support|Lupin: study product **Julia C. Dombrowski, MD, MPH**, Hologic: Grant/Research Support|Mayne Pharmaceuticals: Grant/Research Support

